# Do the pharmacokinetics of statins explain psychiatric symptom improvement from adjunctive statin prescribing in severe mental illness? Three target trial emulation studies

**DOI:** 10.1136/bmjment-2025-302124

**Published:** 2025-12-01

**Authors:** Naomi Launders, Alvin Richards-Belle, Sarah Hardoon, Kenneth Man, Ian C K Wong, David P J Osborn, Joseph F Hayes

**Affiliations:** 1 Division of Psychiatry, University College London, London, UK; 2 Research Department of Practice and Policy, UCL School of Pharmacy, London, UK; 3 Centre for Safe Medication Practice and Research, Department of Pharmacology and Pharmacy, University of Hong Kong Faculty of Medicine, Pokfulam, Hong Kong; 4 Centre for Medicines Optimisation Research and Education, University College London Hospitals NHS Foundation Trust, London, UK; 5 Laboratory of Data Discovery for Health (D24H), New Territories, Hong Kong; 6 Aston Pharmacy School, Aston University, Birmingham, UK; 7 Camden and Islington NHS Foundation Trust, London, UK

**Keywords:** Psychotic Disorders, Schizophrenia, Bipolar and Related Disorders

## Abstract

**Background:**

Evidence regarding the efficacy of adjunct statins to treat severe mental illness (SMI) is mixed. The varying pharmacokinetic properties of statins mean that specific statin-antipsychotic combinations might improve psychiatric symptoms.

**Objective:**

To test whether some statin-antipsychotic/mood stabiliser combinations result in psychiatric symptom improvement in patients with SMI, while others do not, using target trial emulation in observational data.

**Methods:**

We identified patients with SMI (schizophrenia, bipolar disorder, ‘other’ psychoses) prescribed antipsychotics/mood stabilisers and statins from 2000 to 2019 in English linked primary care records (Clinical Practice Research Datalink). We defined hypothetical randomised trials and observational emulations: (1) blood-brain barrier (BBB)-penetrant (simvastatin) versus non-penetrant (atorvastatin/pravastatin/rosuvastatin) statins; (2A) P-glycoprotein inhibitors (simvastatin/atorvastatin) versus non-inhibitors (pravastatin) in patients prescribed aripiprazole/risperidone/olanzapine (P-glycoprotein affinity); (2B) high (aripiprazole/risperidone/olanzapine) versus low (quetiapine) P-glycoprotein affinity antipsychotics in patients prescribed P-glycoprotein-inhibiting statins.

**Findings:**

We found no reduction in our primary outcomes (12-month psychiatric admissions) in trial 1 (HR 1.07, 95% CI 0.88 to 1.31); trial 2A (HR 0.77, 95% CI 0.28 to 2.15); or trial 2B (HR 0.93, 95% CI 0.79 to 1.09). In trial 2B, we observed lower self-harm events (HR 0.60, 95% CI 0.38 to 0.97) in per-protocol analysis and lower psychiatric admissions in the ‘other’ psychoses subgroup (HR 0.53, 95% CI 0.34 to 0.85).

**Conclusions:**

BBB penetrance appears unlikely to be the mechanism by which statins improve SMI symptoms. Interaction with P-glycoprotein may have some effect. Further mechanistic and clinical research is needed to understand statin-antipsychotic interactions and the role of interaction with P-glycoprotein.

**Clinical implications:**

There is currently not enough evidence to guide the prescription of statins for psychiatric symptom improvement in patients with SMI. If there is an effect of statins, it may be through specific statin-antipsychotic combinations.

**WHAT IS ALREADY KNOWN ON THIS TOPIC**

- It has been hypothesised that the anti-inflammatory properties of statins may reduce psychiatric symptoms in people with severe mental illness (SMI), though evidence from both randomised controlled trials and observational studies is mixed.

**WHAT THIS STUDY ADDS**

- We used target trial emulation to conduct three emulated trials to test the hypothesis that the pharmacokinetic properties of statins mean that specific statin-antipsychotic/mood stabiliser combinations would improve psychiatric symptoms in people with SMI. We found no evidence of a role of blood-brain barrier penetrance in psychiatric symptom improvement (reduction in psychiatric admissions or self-harm events), and inconclusive evidence regarding the role of P-glycoprotein-inhibiting statins in combination with antipsychotics that have affinity for P-glycoprotein.

**HOW THIS STUDY MIGHT AFFECT RESEARCH, PRACTICE OR POLICY**

- The results of this study suggest blood-brain barrier penetrance is unlikely to be the mechanism for psychiatric symptom improvement in patients with SMI, whereas further research into the role of P-glycoprotein is warranted. However, the study used clinical data as an indirect measure of mechanistic actions, and further mechanistic and clinical research is needed to test specific antipsychotic-statin interactions. Currently, there is not enough evidence to guide the prescription of statins for symptom improvement in patients with SMI.

## Introduction

 It has been hypothesised that hydroxylmethyl glutaryl coenzyme A reductase inhibitors (statins) may have neuroprotective effects^1^ and improve psychiatric symptoms in people with mental illness.^2^,^5^ However, the results of randomised controlled trials (RCTs) in patients with severe mental illness (SMI; schizophrenia, bipolar disorder and non-organic non-affective psychoses) have been mixed.^36^,^13^ For patients with schizophrenia, two meta-analyses of RCTs reported a modest improvement in the Positive and Negative Syndrome Scale (PANSS) total scores,^2 14^ one found improved total PANSS and negative subscale scores (but not positive subscale scores),^15^ while another found no effect of simvastatin on PANSS.^16^

Some observational studies have found lower risk of psychiatric admissions in people diagnosed with SMI prescribed statins.^4 17^ Another study found statins were associated with lower all-cause mortality, but not suicide.^18^

Given these heterogenous results, potential improvements in mental health may be dependent on combinations of statin and psychotropic medication. Statins that cross the blood-brain barrier (BBB) more readily may have a greater anti-inflammatory, antioxidant and neuroprotective effect in the brain.^1 4^ While simvastatin and atorvastatin are both lipophilic, the smaller molecular weight of simvastatin may aid BBB transfer. Sierra *et al* found that atorvastatin, rosuvastatin and pravastatin do not readily cross the BBB by passive diffusion, while simvastatin does.^1^

A second hypothesised mechanism of action is through improved effectiveness of antipsychotics. The membrane protein P-glycoprotein may be involved in the cellular uptake and efflux of some antipsychotics.^19^ Aripiprazole, risperidone and olanzapine have affinity for the P-glycoprotein in vitro, with higher brain concentrations of these antipsychotics in animal models where P-glycoprotein is absent or inhibited.^19^ Simvastatin and atorvastatin may act as inhibitors of P-glycoprotein, thereby potentially increasing the concentration of antipsychotics such as aripiprazole, risperidone and olanzapine,^20^ and risperidone concentration in humans is higher in the presence of P-glycoprotein inhibitors.^19^

We aimed to test the overarching hypothesis that statin pharmacokinetics result in some statin-antipsychotic/mood stabiliser combinations causing psychiatric symptom improvement in patients with SMI, while others do not. We used target trial emulation (TTE) as a framework to improve the robustness of our study, reducing bias and methodological flaws. We tested three specific questions using rates of psychiatric hospital admissions and self-harm events as proxies for psychiatric symptom deterioration:

1: In patients with SMI prescribed antipsychotics/mood stabilisers, does initiating statins with the most potential to cross the BBB (simvastatin) result in lower psychiatric hospital admissions at 12 months than those initiating atorvastatin, pravastatin or rosuvastatin?

2A: In patients with SMI prescribed antipsychotics with affinity for P-glycoprotein (aripiprazole, risperidone or olanzapine), does initiating statins that inhibit P-glycoprotein (simvastatin or atorvastatin) result in lower psychiatric hospital admissions at 12 months than those initiating pravastatin (no P-glycoprotein inhibition)?

2B: In patients with SMI prescribed statins that inhibit P-glycoprotein (simvastatin or atorvastatin), does initiating antipsychotics with affinity for P-glycoprotein (aripiprazole, risperidone or olanzapine) result in lower psychiatric hospital admissions at 12 months than those initiating quetiapine (no affinity for P-glycoprotein)?

## Methods

### Source population

We used Clinical Practice Research Datalink (CPRD) GOLD and Aurum databases, containing pseudonymised primary care electronic health records (EHRs) from two clinical systems in the UK. CPRD has been shown to be representative of the general UK population in respect to key patient characteristics.^21 22^ We identified a cohort of patients with SMI (schizophrenia, bipolar disorder or other non-organic psychoses) recorded using standardised coding systems (Read or Systematized Nomenclature of Medicine - Clinical Terms (SNOMED CT)) or coding specific to the GP software (EMIS codes) (available at https://github.com/NaomiLaunders/Repurposing-statins-in-SMI). Patients were required to be registered with a primary care practice in England, diagnosed with SMI between 1 January 2000 and 31 December 2019, and between the ages of 18 and 100 at the time of diagnosis. Patients were also required to have received prescriptions for both antipsychotics or mood stabilisers (lithium, sodium valproate or lamotrigine) and statins. CPRD provided linked Hospital Episode Statistics (HES) data, which contain pseudonymised secondary care data in England.

We conducted three TTEs and reported our results in line with both the REporting of studies Conducted using Observational Routinely-collected Data (RECORD)^23^ and TrAnsparent ReportinG of studies Emulating a Target trial (TARGET)^24^ checklists (online supplemental materials). We specified our hypothetical target trial and observational emulations in a prepublished protocol (https://osf.io/hck8n, online supplemental table 1).

#### Trial 1: BBB-penetrant statins (simvastatin) versus non-penetrant statins (atorvastatin, pravastatin or rosuvastatin) in patients with SMI prescribed antipsychotics/mood stabilisers

##### Population

We included patients from our cohort with a diagnosis of SMI prior to statin initiation and at least one prescription for antipsychotics/mood stabilisers at therapeutic dose (online supplemental methods) in the 6 months prior to statin initiation.

We excluded patients who (1) initiated a statin other than simvastatin, atorvastatin, pravastatin or rosuvastatin or who had evidence of prior statin prescriptions, (2) initiated multiple statins across different trial arms on the day of statin initiation, (3) had received injectable antipsychotics in the 3 months prior to statin initiation due to the potential of residual effects of these medications and the incomplete capture of their prescription in primary care and (4) did not have at least 6 months of baseline medical records to reduce the chance of prevalent statin use being recorded as incident statin use (online supplemental figures 1 and 2).

##### Treatment strategies, recruitment period and follow-up

We assigned patients to treatment (simvastatin) and comparator arms (atorvastatin, pravastatin, rosuvastatin) based on the first recorded prescription of the statins of interest between 1 January 2000 and 31 December 2018. Statins were assumed to be at therapeutic dose at initiation. Follow-up began on the day after statin initiation and ended at the earliest of the event of interest, death, EHR end date, 2 years’ follow-up, age 100 or 1 December 2019.

##### Outcomes

Our primary outcome was psychiatric admissions in the 12 months following statin initiation as a proxy for psychiatric symptom deterioration. Secondary outcomes were psychiatric admissions at 3, 6 and 24 months and physical health admissions, accident and injury admissions and self-harm events at 3, 6, 12 and 24 months (outcome definitions: online supplemental methods).

### Trial 2A: P-glycoprotein-inhibiting statins (simvastatin or atorvastatin) versus non-inhibiting statins (pravastatin) in patients with SMI prescribed antipsychotics with affinity for P-glycoprotein (aripiprazole, risperidone or olanzapine)

#### Population

The population for trial 2A was a subset of trial 1 (online supplemental figures 1 and 2), additionally limited to patients initiating simvastatin, atorvastatin or pravastatin; and whose most recent antipsychotic was aripiprazole, risperidone or olanzapine. We excluded those prescribed (1) mood stabilisers only, or never prescribed the study antipsychotics, and (2) high-dose statins in the treatment arm in a post hoc decision as no patients were prescribed high-dose statins in the comparator arm.

#### Treatment strategies, recruitment and follow-up periods and outcomes

We assigned patients to treatment (simvastatin or atorvastatin) and comparator (pravastatin) arms based on the first recorded prescription of the statins of interest. Statins were assumed to be at therapeutic dose at initiation. Recruitment and follow-up periods and outcomes were as described for trial 1.

### Trial 2B: antipsychotics with affinity for P-glycoprotein (aripiprazole, risperidone or olanzapine) versus antipsychotics without affinity (quetiapine) in patients prescribed statins that inhibit P-glycoprotein (simvastatin or atorvastatin)

#### Population

We included patients who initiated aripiprazole, risperidone, olanzapine or quetiapine and who had been prescribed simvastatin or atorvastatin in the 6 months prior to this.

We excluded patients who (1) had evidence of prior prescriptions of the study antipsychotics, but not those previously prescribed other antipsychotic/mood stabilisers, (2) were coprescribed antipsychotics in both the treatment and comparator arms at the date of antipsychotic initiation, (3) had less than 6 months of baseline medical records and (4) received injectable antipsychotics in the 3 months prior to study initiation (online supplemental figures 1 and 2). We did not require patients to have an SMI diagnosis at the time of antipsychotic initiation, and for these patients the SMI subtype was classified as ‘not yet diagnosed’.

#### Treatment strategies, recruitment and follow-up periods and outcomes

We assigned patients to treatment (aripiprazole, risperidone or olanzapine) and comparator (quetiapine) arms based on antipsychotic initiation between 1 January 2000 and 31 December 2018. Follow-up began 1 month after initiation. End of follow-up outcomes were as described in trial 1.

### Confounding

We applied a causal framework to the identification of potential confounders (https://osf.io/hck8n).

We included sex and ethnicity recorded at any point and the following confounders ever recorded prior to study initiation: age at study entry, year of study entry, Index of Multiple Deprivation (IMD) of patient postcode, SMI subtype (schizophrenia, bipolar disorder, other psychoses, not yet diagnosed); time since first SMI diagnosis; time since first antipsychotic/mood stabiliser prescription (trial 1) or first antipsychotic prescription (trials 2A and 2B); cardiovascular disease (diagnosis of hypertension, myocardial infarction or congestive heart failure); diabetes; time since evidence of dyslipidaemia.

We included a count of face-to-face primary care encounters and a binary measure of primary care-recorded self-harm events and prescription of antidepressants (selective serotonin reuptake inhibitors, tricyclic antidepressants or ‘other’) in the 6 months before initiation. We included psychiatric, self-harm, accident and injury, or physical health admissions in the 1 year before initiation (all binary), as well as the most recently recorded total cholesterol level in the 3 years before initiation and body mass index (BMI) value in the 5 years before initiation.

For trial 1, we included the antipsychotic/mood stabiliser prescribed, and in trial 2B, we included time since first statin prescription. Confounders were captured in primary care EHRs, except for hospital admissions which were taken from HES, ethnicity which we extracted from either primary care EHRs or HES and IMD from linked postcode data.

### Missing data

We imputed missing BMI, cholesterol and ethnicity using multiple imputation by chained equations (MICE), with 10 imputed datasets pooled using Rubin’s rules. We included all confounders in the imputation equation as well as 2-year outcomes. We performed MICE separately for each trial and for survival and count models. For imputation used in the Cox regression models, we included the Nelson-Aalen estimator.^25^

We imputed missing statin dose as the median for each statin, and missing patient IMD as the IMD of the patients’ primary care practice.

### Analysis

We used Cox regression, with baseline hazards stratified by primary care practice, as our primary analysis in all trials. We tested the proportional hazard assumption using Schoenfeld residuals and Kaplan-Meier plots. We tested for interactions between trial arms and SMI subtype for the 12-month outcomes, and where significant interactions were found, we presented stratified results. In our secondary analysis, we used negative binomial regression. We assessed all outcomes using an observational analogue of intention-to-treat analysis, with patients analysed in their original treatment arm assignments. All data processing and analysis were performed in R V.4.4.3 and RStudio V.2024.12.1. Scripts are available at https://github.com/NaomiLaunders/Repurposing-statins-in-SMI.

### Prespecified sensitivity analyses

**Figure 1:**
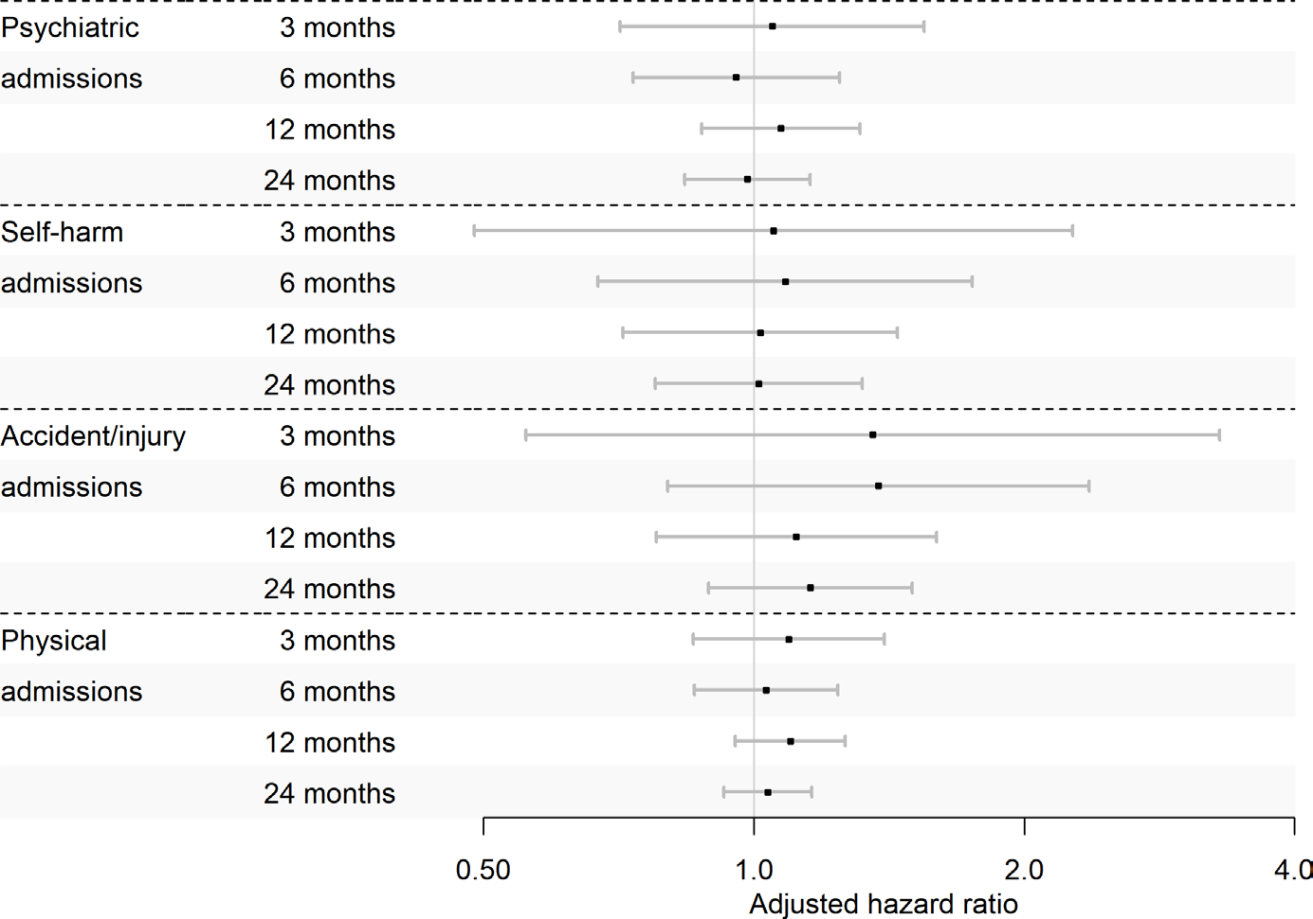
Cox regression results for trial 1: blood-brain barrier-penetrant (simvastatin) versus non-penetrant (atorvastatin, pravastatin or rosuvastatin) statins in patients with severe mental illness (SMI) prescribed antipsychotics or mood stabilisers.

**Figure 2:**
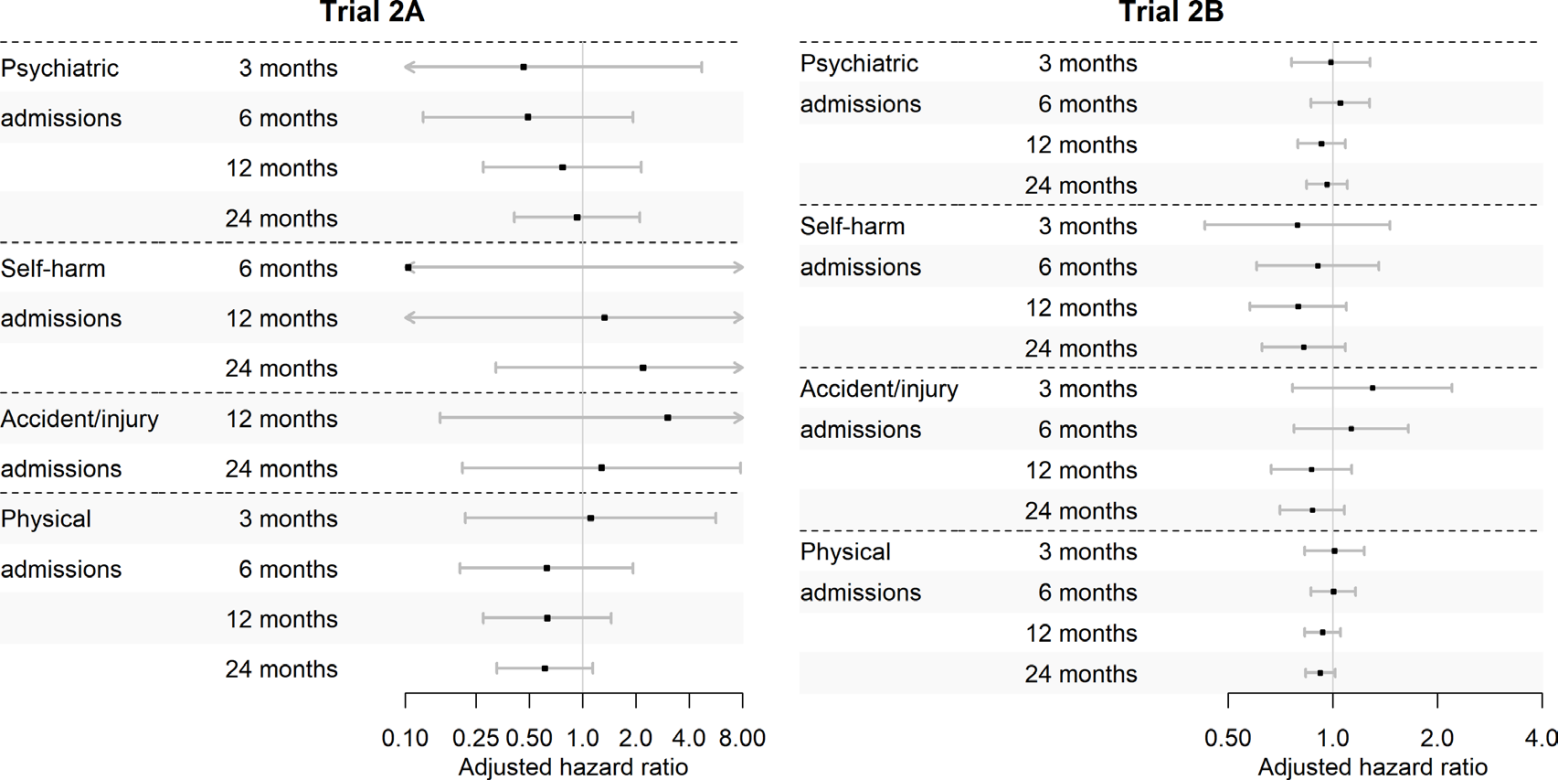
Cox regression results. Trial 2A: P-glycoprotein-inhibiting (simvastatin or atorvastatin) versus non-inhibiting (pravastatin) statins in patients with severe mental illness (SMI) prescribed antipsychotics with P-glycoprotein affinity (aripiprazole, risperidone or olanzapine). Trial 2B: antipsychotics with P-glycoprotein affinity (aripiprazole, risperidone or olanzapine) versus no affinity (quetiapine) in patients prescribed statins that inhibit P-glycoprotein (simvastatin or atorvastatin).

We reran the primary analyses using (1) an observational analogue of a per-protocol effect and (2) inverse probability weighting (IPW) to control for confounders (online supplemental methods and online supplemental methods figures 1–3). We did not perform our prespecified analysis using prevalent statin prescriptions as this only yielded an extra 19 patients in the smallest arm of trial 2A.

## Findings

We identified 72 096 adults in our overall cohort, of which 19 033 were eligible for trial 1; 6081 for trial 2A; and 9398 for trial 2B (table 1, online supplemental table 2, online supplemental figures 1 and 2). The proportional hazard assumption held for all 12-month outcomes in all three trials (online supplemental figures 3–5).

**Table 1:** Patient characteristics for three target trial emulation studies

Arm		Trial 1		Trial 2A		Trial 2B	
		Comparator,* n=6703	Treatment,† n=12 330	Comparator,‡ n=100	Treatment,§ n=5981	Comparator,¶ n=2645	Treatment,** n=6753
Age at index (median (IQR))		56.00 (48.00, 66.00)	57.00 (47.00, 66.00)	54.00 (46.00, 65.25)	54.00 (46.00, 64.00)	65.00 (54.00, 76.00)	66.00 (55.00, 76.00)
Sex (%)	Female	3269 (48.77)	5985 (48.54)	49 (49.00)	2566 (42.90)	1459 (55.16)	3634 (53.81)
	Male	3434 (51.23)	6345 (51.46)	51 (51.00)	3415 (57.10)	1186 (44.84)	3119 (46.19)
Region (%)	E Midlands	148 (2.21)	260 (2.11)	<5	111 (1.86)	60 (2.27)	141 (2.09)
	East England	268 (4.00)	561 (4.55)	5 (5.00)	243 (4.06)	141 (5.33)	257 (3.81)
	London	1505 (22.45)	2598 (21.07)	35 (35.00)	1492 (24.95)	451 (17.05)	1531 (22.67)
	North East	245 (3.66)	543 (4.40)	<5	269 (4.50)	98 (3.71)	272 (4.03)
	North West	1639 (24.45)	2724 (22.09)	36 (36.00)	1306 (21.84)	689 (26.05)	1360 (20.14)
	South East	1103 (16.46)	2200 (17.84)	8 (8.00)	1007 (16.84)	511 (19.32)	1193 (17.67)
	South West	581 (8.67)	1227 (9.95)	5 (5.00)	498 (8.33)	210 (7.94)	740 (10.96)
	W Midlands	979 (14.61)	1810 (14.68)	7 (7.00)	870 (14.55)	429 (16.22)	1013 (15.00)
	Y&H	235 (3.51)	407 (3.30)	<5	185 (3.09)	56 (2.12)	246 (3.64)
Ethnicity (%)	Asian	480 (7.16)	766 (6.21)	6 (6.00)	509 (8.51)	162 (6.12)	577 (8.54)
	Black	356 (5.31)	577 (4.68)	11 (11.00)	397 (6.64)	68 (2.57)	403 (5.97)
	Mixed	105 (1.57)	142 (1.15)	<5	86 (1.44)	27 (1.02)	76 (1.13)
	Other	86 (1.28)	154 (1.25)	<5	90 (1.50)	32 (1.21)	79 (1.17)
	White	5613 (83.74)	10 561 (85.65)	76 (76.00)	4855 (81.17)	2333 (88.20)	5558 (82.30)
	Missing	63 (0.94)	130 (1.05)	<5	44 (0.74)	23 (0.87)	60 (0.89)
SMI diagnosis (%)	Bipolar disorder	2904 (43.32)	5495 (44.57)	25 (25.00)	1495 (25.00)	1000 (37.81)	1626 (24.08)
	Other psychoses	1182 (17.63)	1918 (15.56)	22 (22.00)	1356 (22.67)	670 (25.33)	2146 (31.78)
	Schizophrenia	2617 (39.04)	4917 (39.88)	53 (53.00)	3130 (52.33)	321 (12.14)	1461 (21.63)
	Not yet diagnosed					654 (24.73)	1520 (22.51)

Counts less than 5 have been redacted to avoid accidental disclosure of patient details.

* Patients on any antipsychotic/mood stabiliser initiating atorvastatin, pravastatin or rosuvastatin (BBB non-penetrant).

† Patients on any antipsychotic/mood stabiliser initiating simvastatin (BBB penetrant).

‡ Patients on aripiprazole, risperidone or olanzapine initiating pravastatin (no inhibition of P-glycoprotein).

§ Patients on aripiprazole, risperidone or olanzapine initiating simvastatin or atorvastatin (P-glycoprotein inhibitor).

¶ Patients on simvastatin or atorvastatin initiating quetiapine (no P-glycoprotein affinity).

** Patients on simvastatin or atorvastatin initiating aripiprazole, risperidone or olanzapine (P-glycoprotein affinity).

BBB, blood-brain barrier; E, East; SMI, severe mental illness; W, West; Y&H, Yorkshire and the Humber.

### Trial 1: BBB-penetrant statins (simvastatin) versus non-penetrant statins (atorvastatin, pravastatin, rosuvastatin) in patients with SMI prescribed antipsychotics/mood stabilisers

#### Primary outcome: psychiatric hospital admissions at 12 months

We found no evidence of lower psychiatric admissions in people with SMI prescribed BBB-penetrant versus non-penetrant statins at 12 months in our adjusted analysis (adjusted HR (aHR) 1.07, 95% CI 0.88 to 1.31; table 2, figure 1). Prior to adjustment, those prescribed simvastatin had a higher risk of psychiatric admissions (online supplemental table 3; HR 1.22, 95% CI 1.07 to 1.39). We found no evidence of interaction effects between SMI diagnosis and trial arm for any 12-month outcomes.

**Table 2:** Unadjusted and adjusted Cox regression results for the three target trial emulation studies

	3 months: adjusted HR (95% CI)	6 months: adjusted HR (95% CI)	12 months: adjusted HR (95% CI)	24 months: adjusted HR (95% CI)
Trial 1: blood-brain barrier-penetrant (simvastatin) versus non-penetrant (atorvastatin, pravastatin or rosuvastatin) statins in patients with SMI prescribed antipsychotics or mood stabilisers				
Psychiatric admissions	1.05 (0.71 to 1.55)	0.96 (0.73 to 1.25)	1.07 (0.88 to 1.31)	0.98 (0.84 to 1.16)
Self-harm events	1.05 (0.49 to 2.26)	1.08 (0.67 to 1.75)	1.02 (0.72 to 1.44)	1.01 (0.78 to 1.32)
Physical health admissions	1.09 (0.86 to 1.40)	1.03 (0.86 to 1.24)	1.10 (0.95 to 1.26)	1.04 (0.93 to 1.16)
Accident/injury admissions	1.36 (0.56 to 3.30)	1.38 (0.80 to 2.36)	1.12 (0.78 to 1.60)	1.16 (0.89 to 1.50)
Trial 2A: P-glycoprotein-inhibiting (simvastatin or atorvastatin) versus non-inhibiting (pravastatin) statins in patients with SMI prescribed antipsychotics with P-glycoprotein affinity (aripiprazole, risperidone or olanzapine)				
Psychiatric admissions	0.46 (0.05 to 4.70)	0.49 (0.13 to 1.92)	0.77 (0.28 to 2.15)	0.93 (0.41 to 2.10)
Self-harm events	–	0.10 (0.00 to 34.41)	1.32 (0.02 to 71.14)	2.19 (0.32 to 14.79)
Physical health admissions	1.11 (0.22 to 5.65)	0.63 (0.20 to 1.93)	0.63 (0.27 to 1.45)	0.61 (0.33 to 1.14)
Accident/injury admissions	–	–	3.02 (0.16 to 57.75)	1.28 (0.21 to 7.78)
Trial 2B: antipsychotics with P-glycoprotein affinity (aripiprazole, risperidone or olanzapine) versus no affinity (quetiapine) in patients prescribed statins that inhibit P-glycoprotein (simvastatin or atorvastatin)				
Psychiatric admissions	0.99 (0.76 to 1.28)	1.05 (0.87 to 1.28)	0.93 (0.79 to 1.09)	0.96 (0.84 to 1.10)
Self-harm events	0.79 (0.43 to 1.46)	0.90 (0.60 to 1.35)	0.80 (0.58 to 1.09)	0.82 (0.63 to 1.09)
Physical health admissions	1.01 (0.83 to 1.23)	1.00 (0.87 to 1.16)	0.93 (0.83 to 1.05)	0.92 (0.83 to 1.01)
Accident/injury admissions	1.30 (0.77 to 2.20)	1.13 (0.77 to 1.65)	0.87 (0.67 to 1.13)	0.87 (0.71 to 1.08)

SMI, severe mental illness.

#### Secondary outcomes

We found no evidence of differences in any secondary outcomes in adjusted analyses (table 2, figure 1); however, the psychiatric admission rate was higher in those initiating a BBB-penetrant statin than in the comparator arm at 3, 6 and 24 months in the unadjusted analysis (online supplemental table 3).

#### Negative binomial models and sensitivity analyses

The negative binomial models agreed with the Cox regression analysis, except for self-harm events at 3 months which were elevated in those initiating a BBB-penetrant statin compared with those prescribed a non-penetrant statin (adjusted incidence rate ratio (aIRR) 1.95, 95% CI 1.03 to 3.70; online supplemental table 4). The IPW model found higher rates of psychiatric admissions and self-harm events at 3 months in those initiating a BBB-penetrant statin and more accident/injury admissions at 6 months (online supplemental table 5). As statin dose remained unbalanced after IPW (online supplemental methods figure 2), we ran the IPW regression additionally adjusting for this; however, this did not change the results. The per-protocol analysis agreed with the main results (online supplemental table 6).

### Trial 2A: P-glycoprotein-inhibiting statins (simvastatin, atorvastatin) versus non-inhibiting statins (pravastatin) in patients with SMI prescribed antipsychotics with affinity for P-glycoprotein (aripiprazole, risperidone, olanzapine)

#### Primary outcome: psychiatric hospital admissions at 12 months

In patients prescribed antipsychotics with affinity for P-glycoprotein, there was no evidence of a difference in psychiatric admissions at 12 months between patients initiating P-glycoprotein-inhibiting or non-inhibiting statins (aHR 0.77, 95% CI 0.28 to 2.15; table 2, figure 2). However, in both analyses, we found a large negative effect size with wide CIs. Small numbers in the comparator arm (n=100) meant that we were unable to test for interactions between trial arm and SMI diagnosis.

#### Secondary outcomes

We observed no difference in secondary outcomes at any time point (table 2, figure 2). We were unable to model accident/injury admissions at 3 or 6 months or self-harm admissions at 3 months due to the low number of outcome events.

#### Negative binomial models and sensitivity analyses

The negative binomial models agreed with the Cox models for all outcomes (online supplemental table 4). The IPW model found higher rates of physical health admission at 3, 6 and 12 months in those initiating P-glycoprotein-inhibiting statins (online supplemental table 5). However, the CIs were wide, and the IPW failed to balance covariates (online supplemental methods), resulting in a very small effective population size. The per-protocol analysis agreed with the main analysis (online supplemental table 6).

### Trial 2B: antipsychotics with affinity for P-glycoprotein (aripiprazole, risperidone, olanzapine) versus antipsychotics without affinity (quetiapine) in patients prescribed statins that inhibit P-glycoprotein (simvastatin, atorvastatin)

#### Primary outcome: psychiatric hospital admissions at 12 months

In patients prescribed statins that inhibit P-glycoprotein, there was no difference in psychiatric admissions at 12 months between those initiating antipsychotics with P-glycoprotein affinity compared with those initiating antipsychotics without affinity (aHR 0.93, 95% CI 0.79 to 1.09; table 2, figure 2). We found a significant interaction between 12 months’ psychiatric admissions and SMI diagnosis (Wald test: p=0.002). In stratified analysis, patients with other psychoses initiating antipsychotics with affinity for P-glycoprotein had a lower rate of psychiatric admissions than the comparator arm (aHR 0.53, 95% CI 0.34 to 0.85; online supplemental figure 6). For those with a diagnosis of schizophrenia, we found a large negative effect size, though CIs included the null (aHR 0.60, 95% CI 0.33 to 1.09).

#### Secondary outcomes

We observed no differences in any adjusted analyses of secondary outcomes (table 2, figure 2), though in unadjusted analysis self-harm events were less frequent in patients initiating antipsychotics with affinity for P-glycoprotein compared with those initiating antipsychotics without affinity at 12 and 24 months (online supplemental table 3). We found no evidence of an interaction between SMI diagnosis and trial arm for accident/injury admissions or self-harm events but did for physical health (Wald test: p=0.010) admissions. In stratified analysis, patients with schizophrenia initiating antipsychotics with affinity for P-glycoprotein had a lower rate of physical health admissions than the comparator arm (aHR 0.50, 95% CI 0.31 to 0.82; online supplemental figure 6).

#### Negative binomial models and sensitivity analyses

Negative binomial models found lower physical health admissions at 24 months in those initiating antipsychotics with affinity for P-glycoprotein compared with the comparator arm (aIRR 0.90, 95% CI 0.81 to 0.99) and evidence of a lower rate of psychiatric admissions at 24 months, though the CI was close to the null (aIRR 0.87, 95% CI 0.76 to 0.999; online supplemental table 4). In contrast, the IPW model found no significant differences across outcomes (online supplemental table 5). In per-protocol analysis, those initiating antipsychotics with affinity for P-glycoprotein had a lower rate of self-harm events at 12 months compared with the comparator arm (HR 0.60, 95% CI 0.38 to 0.97; online supplemental table 6).

## Discussion

We conducted three TTE studies to test whether the pharmacokinetic properties of statins were implicated in psychiatric symptom improvement in patients with SMI.

Despite simvastatin having the most potential to cross the BBB based on molecular weight and lipophilicity, and the most BBB penetrance in vitro,^1^ we found no reduction in psychiatric admissions in patients prescribed simvastatin versus other statins. Most RCTs of adjunct statins have focused on simvastatin, and while some have found weak evidence of an improvement in some measures of symptoms, the findings have been mixed.^36^,^8 12^

In secondary and sensitivity analyses, we found some evidence of elevated psychiatric admissions and self-harm events at 3 months in those prescribed simvastatin. This is in line with Postolache *et al*, who found higher rates of psychiatric admissions in patients prescribed statins that readily crossed the BBB. They postulated that BBB-penetrant statins may lower cholesterol in the brain, thereby affecting serotonin synthesis and increasing cognitive dysfunction, impulsivity and anxiety.^4^ While our findings were not in our main analyses and should therefore be interpreted cautiously, they require further investigation.

Our findings regarding P-glycoprotein were inconclusive. We found no evidence that statins that inhibit P-glycoprotein reduce psychiatric admissions compared with those that do not in patients prescribed antipsychotics with affinity for P-glycoprotein. However, this analysis was statistically underpowered due to the low prevalence of pravastatin prescribing.

We did find some evidence in stratified and sensitivity analyses that in patients who were prescribed P-glycoprotein-inhibiting statins, those initiating antipsychotics with P-glycoprotein affinity had lower rates of psychiatric admissions or self-harm events compared with those initiating antipsychotics without affinity. In stratified analysis, patients with a diagnosis of ‘other psychoses’ and prescribed P-glycoprotein-inhibiting statins and initiating antipsychotics with affinity for P-glycoprotein also had lower rates of psychiatric admissions at 12 months than those initiating quetiapine. The effect size was similar in the schizophrenia group, but CIs were wide and contained the null. In our per-protocol sensitivity analysis, patients initiating antipsychotics with affinity for P-glycoprotein also had lower rates of self-harm at 12 months compared with those initiating quetiapine.

An interaction between P-glycoprotein-inhibiting statins and antipsychotics with affinity for P-glycoprotein may explain the inconclusive results of published simvastatin trials and in our trial 1. Two RCTs that included patients on any antipsychotic agent, with simvastatin as an adjunct, found no change in admissions at 12 months,^3 12^ whereas RCTs limited to patients prescribed risperidone (which has affinity for P-glycoprotein) with or without simvastatin or atorvastatin (P-glycoprotein inhibitors) have found improvements in negative symptoms.^6 11^

Taken together, our results suggest that the psychiatric mechanism of action of statins is unlikely to be primarily via BBB penetrance. P-glycoprotein inhibition increasing the effects of certain antipsychotics is a possible mechanism of action, though cautious interpretation is required, as is further mechanistic and clinical research to clarify the role of P-glycoprotein.

### Strengths and limitations

We used large representative EHRs and TTE to estimate the causal effect of statin/psychotropic combinations on psychiatric admissions in patients with SMI.

The observational design of the study allows more combinations of statins and antipsychotics/mood stabilisers to be tested, with longer outcomes and larger population sizes than would be possible in an RCT, and provides evidence for targeting RCTs at likely candidates for psychiatric symptom improvement in patients with SMI. While observational data are vulnerable to issues of bias and confounding, we took steps to reduce these by pre-registering our protocol, designing a hypothetical RCT to emulate the trial and using both traditional confounder adjustment and IPW approaches. The head-to-head design of our study reduces the likelihood of confounding by indication compared with studies that contrast against non-prescription. However, unmeasured and residual confounding cannot be ruled out and the lack of randomisation may have introduced bias in our study. Further approaches such as double-robust estimation^26^ may have further minimised confounding but were outside the scope of this work.

Our strict inclusion/exclusion criteria limited the size and generalisability of our cohort. They were older than other cohorts of patients with SMI, and our analysis was underpowered to investigate some combinations of statins and antipsychotics, particularly in trial 2A. For trials 1 and 2B, the sample size was greater than previous RCTs, though our null finding could still be due to lack of power. Additionally, we did not account for multiple testing in our analysis.

We were limited in our ability to capture psychiatric symptoms in EHRs. We used a reduction in psychiatric admissions and self-harm events as a measure of psychiatric symptom improvement, in line with previous studies.^4 17^ However, this is a crude proxy for true symptom improvement and does not capture smaller changes in mental health status. Therefore, there may have been symptom changes between the treatment arms which we were unable to detect, such as changes in cognition, functioning or mood symptoms.^1 5^ Furthermore, while psychiatric hospital admissions will be well captured in EHRs, self-harm events may be less reliably captured.^27^

There is likely misclassification of patients who were prescribed but did not take medication. Further, we converted antipsychotic dose to olanzapine equivalents and imputed statin dose where it was absent, both of which may have introduced error. However, since all patients had to be on both antipsychotics and statins, the differences are unlikely to be differential between treatment and comparator arms. Statins were assumed to be at therapeutic dose at initiation. Some patients may have initiated statins at a subtherapeutic dose, which may have resulted in an underestimation of effectiveness. However, we accounted for differences in starting dose in our analysis and therefore this assumption will have had a limited effect on the comparative effectiveness of statins between study arms. There are many factors that influence the frequency and recording of self-harm events and psychiatric admissions; however, it is unlikely that these factors were differential across treatment and comparator arms.

We found evidence of reduced self-harm events and psychiatric admissions following initiation of antipsychotics with affinity for P-glycoprotein in patients prescribed P-glycoprotein-inhibiting statins. However, these results were not our primary analysis. Furthermore, while a recent study found no differences in psychiatric admissions between patients prescribed these four antipsychotics,^28^ we cannot rule out the possibility that these results are due to differences in the effectiveness of these antipsychotics, rather than the combination of antipsychotic plus statin.

We used clinical data to test hypothesised mechanisms of action of statins and antipsychotics/mood stabilisers. However, this is an indirect measure of the mechanisms of action, so it requires cautious interpretation. Furthermore, while we formed our hypotheses based on previous work, the mechanisms of action of both statins and antipsychotics are not fully elucidated, and it is possible that certain statin-antipsychotic/mood stabiliser combinations may be effective through mechanisms we did not consider. For example, an RCT of pravastatin, which does not readily cross the BBB and does not have affinity for P-glycoprotein, also found a small reduction in positive symptoms at 6 weeks in patients with schizophrenia,^9^ perhaps suggesting that alternative mechanisms may play a role.

We were unable to investigate the effects of individual statin-antipsychotic/mood stabiliser combinations. Additionally, simvastatin possesses both BBB-penetrant and P-glycoprotein inhibitory properties, meaning we could not separate these mechanisms completely. Despite this, in trial 1, any observed effect of simvastatin is likely to be due to BBB penetrance, as the trial was not limited to antipsychotics with affinity for P-glycoprotein, and atorvastatin in the comparator arm also inhibits P-glycoprotein.

### Clinical implications

In secondary and subgroup analyses, we found weak evidence that for those prescribed aripiprazole, risperidone or olanzapine, the addition of simvastatin or atorvastatin may reduce psychiatric admissions and self-harm. However, we also found weak evidence of higher psychiatric admissions in those prescribed simvastatin versus other statins. Despite these findings, there is currently not enough evidence to guide the prescription of specific statins for symptom improvement in patients with SMI.

Our results suggest a need for RCTs focusing on individual statin-antipsychotic/mood stabiliser combinations, particularly statins and antipsychotics that interact with P-glycoprotein, and further studies into the mechanisms of action and interaction of statins and antipsychotics, and effectiveness in specific subgroups of individuals with SMI.

## Supplementary material

10.1136/bmjment-2025-302124 online supplemental file 1

## Data Availability

No data are available.
